# The crystal structure of human dopamine β-hydroxylase at 2.9 Å resolution

**DOI:** 10.1126/sciadv.1500980

**Published:** 2016-04-08

**Authors:** Trine V. Vendelboe, Pernille Harris, Yuguang Zhao, Thomas S. Walter, Karl Harlos, Kamel El Omari, Hans E. M. Christensen

**Affiliations:** 1 Department of Chemistry, Kemitorvet 207, Technical University of Denmark, DK-2800 Kgs. Lyngby, Denmark.; 2 Division of Structural Biology, Wellcome Trust Centre for Human Genetics, University of Oxford, Oxford OX3 7BN, UK.

**Keywords:** Life sciences, structural biology, biochemistry, biomolecules, norepinephrine, dopamine, crystal structure, dopamine β-hydroxylase, neurochemistry

## Abstract

The norepinephrine pathway is believed to modulate behavioral and physiological processes, such as mood, overall arousal, and attention. Furthermore, abnormalities in the pathway have been linked to numerous diseases, for example hypertension, depression, anxiety, Parkinson’s disease, schizophrenia, Alzheimer’s disease, attention deficit hyperactivity disorder, and cocaine dependence. We report the crystal structure of human dopamine β-hydroxylase, which is the enzyme converting dopamine to norepinephrine. The structure of the DOMON (dopamine β-monooxygenase N-terminal) domain, also found in >1600 other proteins, reveals a possible metal-binding site and a ligand-binding pocket. The catalytic core structure shows two different conformations: an open active site, as also seen in another member of this enzyme family [the peptidylglycine α-hydroxylating (and α-amidating) monooxygenase], and a closed active site structure, in which the two copper-binding sites are only 4 to 5 Å apart, in what might be a coupled binuclear copper site. The dimerization domain adopts a conformation that bears no resemblance to any other known protein structure. The structure provides new molecular insights into the numerous devastating disorders of both physiological and neurological origins associated with the dopamine system.

## Teaser

This first structure of the enzyme converting dopamine to norepinephrine provides new perspectives on numerous disorders.

## INTRODUCTION

Dopamine β-hydroxylase (EC 1.14.17.1, dopamine β-monooxygenase) (DBH) catalyzes the hydroxylation of dopamine to norepinephrine (*1*) and is thus vital for regulation of these neurotransmitters. The norepinephrine pathway, the only source of norepinephrine and epinephrine, is believed to modulate many behavioral and physiological processes, such as mood, overall arousal, attention, and sexual behavior (*2*), as well as stress (*3*), learning, and memory (*4*). The level of and balance between dopamine and norepinephrine are implicated in a large number of diseases of both physiological, neurological, and psychiatric character, such as hypertension [ranked as the world’s largest disease burden (*5*)], congestive heart failure (*6*), Alzheimer’s disease (*7*), and drug addiction (*8*), as well as Parkinson’s disease, Huntington’s chorea, Tourette syndrome, depression, and attention deficit hyperactivity disorder (ADHD). For review, see the study by Cubells and Zabetian (*9*).

DBH is a member of a small unique class of copper-containing hydroxylases that are found in eukaryotes, and all play a critical role in the biosynthesis of neurotransmitters and hormones. The other members of the family are the bifunctional enzyme peptidylglycine α-hydroxylating (and α-amidating) monooxygenase (PHM) (*10*, *11*), monooxygenase X (DBH-like monooxygenase protein 1, MOXD1) (*12*), and tyramine β-monooxygease (TBH) (*13*), which is the insect homolog of DBH.

The overall domain alignment of this class of copper-containing hydroxylases is provided in fig. S1. They are all multidomain enzymes with a common catalytic core fused to different types of domains. DBH has an N-terminal DOMON (dopamine β-monooxygenase N-terminal) domain, which belongs to the class of DOMON-like domains (*14*). The DOMON domain class is divided into at least nine families that are distantly related by amino acid sequences (*14*). The function of DOMON domains is largely unknown (*14*), but they are involved in ligand binding, either as heme- or sugar-binding domains (*14*). The catalytic core of DBH shows high sequence homology (see fig. S2) with the catalytic core of PHM (*15*) (PHMcc). It consists of two domains, the Cu_H_ and Cu_M_ domains, each binding one copper. Finally, at the C terminus, there is an approximately 100-residue domain with no sequence resemblance to any known domains (*16*), which is referred to as a dimerization domain. DBH is seen both as a homodimer and as a homotetramer. DBH contains 15 cysteine residues, of which many are conserved between DBH of different organisms (see fig. S3). On the basis of studies of bovine DBH, 14 cysteines are involved in disulfide bridge formation, 6 are intramolecular bonds, and 2 are intermolecular bonds (*17*).

DBH is an ascorbate-dependent glycoprotein (*18*) that requires two type 2 bound copper ions per subunit to be active (*19*, *20*). The copper sites are labile (*20*) and termed Cu_H_ and Cu_M_, respectively. Cu_H_ is coordinated to three histidines and Cu_M_ to two histidines and a methionine. On the basis of spectroscopic studies (*21*) and structural studies of PHMcc (*11*), it is suggested that Cu_M_ is involved in dioxygen binding and is the site for substrate hydroxylation, and that Cu_H_ is the site of electron transfer (*22*). During the reaction, an O atom from molecular O_2_ is inserted at the β-carbon in dopamine with retention of configuration, and the second O atom goes to water. The reaction also requires two electrons provided by two ascorbate molecules (*23*) that are oxidized to semihydroascorbate (*24*). In the known structures of this class of enzymes, the two copper ions are more than 11 Å apart and exposed to solvents (*11*). Despite vast investigations [for reviews, see the studies by Osborne and Klinman (*25*) and by Solomon *et al*. (*26*)], it is not entirely clear how these tightly coupled reactions occur (*25*, *26*).

Here, we report the first crystal structure of DBH: the structure of full-length dimeric human DBH.

## RESULTS

### Overall structure

DBH expressed in human embryonic kidney (HEK) 293S cells is present both as a dimer and a tetramer, which can be separated by size exclusion chromatography. The dimer and tetramer do not interconvert in the pH interval 4 to 9 (see figs. S4 to S7). However, under denaturing conditions, the tetramer converts to a dimer, and upon addition of a reducing agent, the dimer converts to a monomer (see fig. S8). Crystallization experiments gave diffraction quality crystals of the dimeric form.

**Fig. 1:**
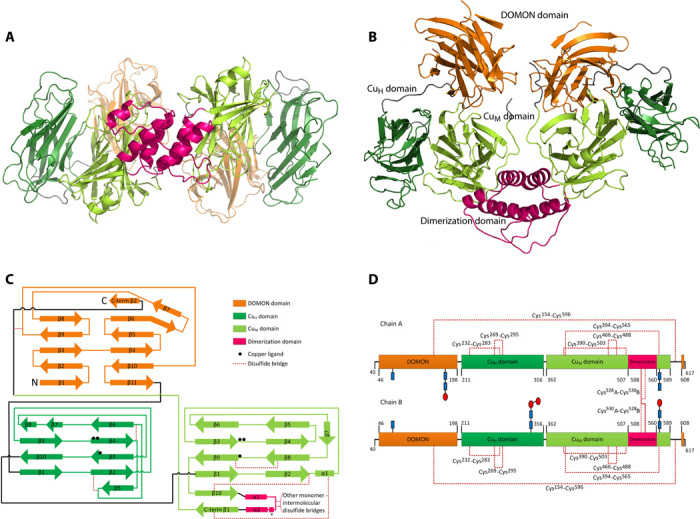
Structure of the human DBH dimer. (**A** and **B**) Overall structure seen from two angles (90° to each other). The DOMON domain is displayed in orange, the Cu_H_ domain in dark green, the Cu_M_ domain in light green, and the dimerization domain in magenta. The interdomain regions are in gray. (**C**) Secondary structure organization of DBH. The black spheres represent positions of the copper ligands. The α helix marked “*” in the dimerization domain is only seen in chain A. A detailed list of secondary structure assignment is provided in table S1. C-term, C-terminal. (**D**) Disulfide bridge pattern in the DBH dimer. The Cu_H_ domain contains two disulfide bridges. The Cu_M_ domain contains two disulfide bridges and forms an additional one with the dimerization domain. The DOMON domain and the dimerization domain are linked via C^154^-C^596^. Chain A is linked via two intermolecular disulfide bonds with chain B in the dimerization domain. Glycosylation is observed at all four predicted sites: Asn^64^, Asn^184^, Asn^344^, and Asn^566^. Glycosylation clearly observed in the electron density map (see figs. S10 and S11) is shown, with *N*-acetylglucosamine as blue rectangles and mannose as red ovals. The position of the disulfide bridges and the glycosylation on the three-dimensional structure is shown in fig. S12.

The overall three-dimensional structure of dimeric human DBH is shown in Fig. 1, and the overall architecture of the fold is shown in Fig. 1C. Each chain folds into four domains: the DOMON domain, the catalytic Cu_H_ and Cu_M_ domains, and the C-terminal dimerization domain.

The DOMON domain has an immunoglobulin (Ig)–like β-sandwich structure, the catalytic core (the Cu_H_ and Cu_M_ domains) has the same topology as the structure of PHM (*11*), and the dimerization domains consisting of two antiparallel α helices form a four-helix bundle. Following the dimerization domain, there is a β-strand (residues 561 to 566) taking part in the catalytic Cu_M_ domain and a β-strand (residues 608 to 614) that is part of the DOMON domain, creating a very integrated structure. This is illustrated in the secondary structure organization of DBH shown in Fig. 1 (C and D) and in fig. S9.

**Fig. 2:**
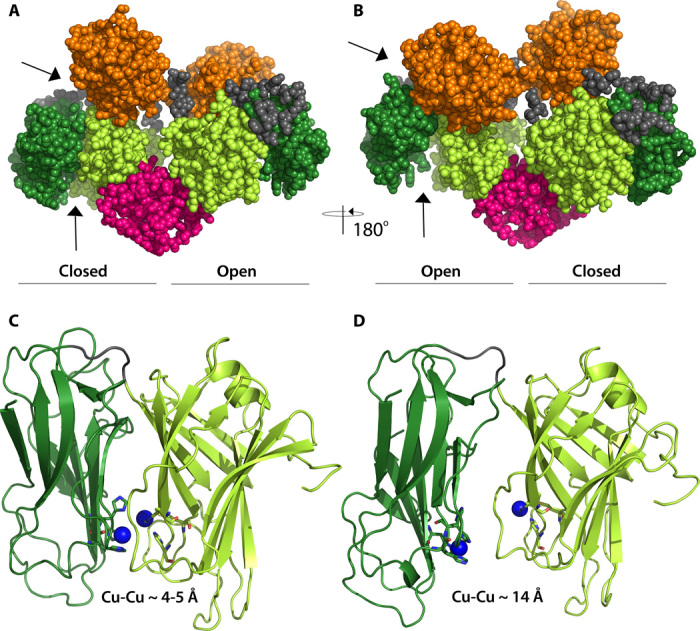
The two conformations of the DBH catalytic core reveal a closed and an open active site. (**A**) Same orientation as Fig. 1B with chain A to the left. (**B**) View from the back, with chain A to the right. (**C**) Closed conformation of the catalytic domain as seen in chain A. (**D**) Open conformation of the catalytic domain as seen in chain B. Cu_M_ in chain A is modeled in the structure, whereas the three other coppers are inserted manually in a position indicated by the position of the conserved active site ligands. Same color coding as in Fig. 1.

The dimeric structure is asymmetric. In the A chain, the two catalytic Cu_H_ and Cu_M_ domains are in a closed conformation, and in the B chain, they adopt the same open conformation as seen in PHM. As evident from Fig. 1 (A and B) and Fig. 2 (A and B), the catalytic Cu_H_ domain in chain A is moved away from the DOMON domain and closer to the catalytic Cu_M_ domain. The asymmetry is also reflected in the dimerization domain, where an extra α helix is seen in the A chain. It should also be noted that although the overall conformation is quite different, the individual domains in the two molecules align nicely, except for the dimerization domain. Alignment of the Cα’s of DOMON A on DOMON B gives a root mean square deviation (RMSD) of 0.71 Å for 149 atoms, alignment of the catalytic Cu_H_ domain A on Cu_H_ B gives an RMSD of 1.15 Å for 137 atoms, and alignment of the catalytic Cu_M_ domain A on Cu_M_ B gives an RMSD of 0.69 Å for 169 atoms. The dimerization domain does, however, not overlay very well with itself (RMSD of 3.83 Å for 53 atoms). Omitting the loop/helix from residues 525 to 538 improves the alignment, and the RMSD becomes 0.87 Å.

### DOMON domain

**Fig. 3:**
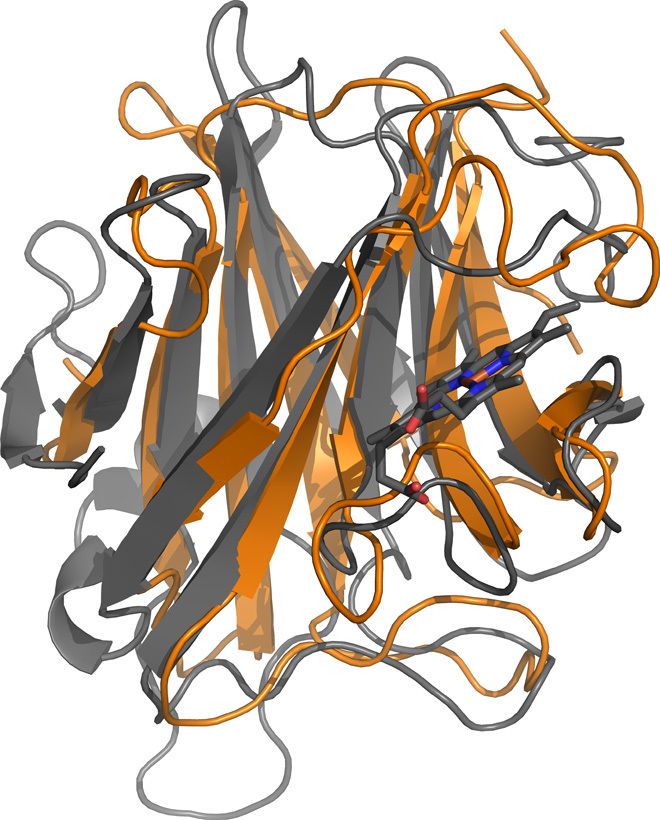
Alignment of the DBH DOMON domain with the cytochrome domain of CDH. DOMON (chain A) is shown in orange and the cytochrome domain of CDH in gray (PDB ID 1D7B). The heme group in CDH is shown in ball-and-stick. The RMSD is 2.53 Å for the backbone atoms, indicating an identical fold of the two domains.

The DOMON superfamily structure is an Ig-like β-sandwich with 10 to 11 β-strands and a ligand-binding pocket (*14*). Here, we present the first experimental structure of the DBH DOMON domain. In DBH, the core structure of the DOMON domain (residues 46 to 198) folds up in a crescent-like structure consisting of two β sheets in a β*-*sandwich, containing five and six antiparallel β-strands, respectively, as shown in Fig. 1 (A to C). The C-terminal sheet includes a β-strand (residues 608 to 614) following the dimerization domain (see Fig. 1). A Dali server search shows that the overall fold of the DBH DOMON domain is identical to the cytochrome domain of white root fungus *Phanerochaete* *chrysosporium* cellobiose dehydrogenase (CDH) [Protein Data Bank (PDB) ID 1D7B] and the *Aromatoleum aromaticum* ethylbenzene dehydrogenase α subunit (PDB ID 2IVF) and, to a lesser extent, the carbohydrate-binding module from *Thermotoga maritima* xylanase (10ACBM9-2) (PDB ID 1I8A). Structural alignment of the DOMON domain in DBH with the cytochrome domain of CDH shows an identical fold of the two domains (see Fig. 3). The DOMON domains in CDH and in the xylanase carbohydrate-binding module bind a heme group and a sugar, respectively. However, a search for binding pockets using the CASTp (Computed Atlas of Surface Topography of proteins) server does not reveal any binding pockets in that area in the DBH DOMON domain—it is too narrow and partially closed by the loop made by residues 173 to 188. Moreover, no typical heme axial ligands (methionine, histidine, lysine, and cysteine) are present, nor are the tryptophan residues binding the sugar in the xylanase carbohydrate-binding module observed. However, from the structural alignment in Fig. 3, it is obvious that there could easily be made room for binding of a small molecule in the DBH DOMON domain at the exterior of the C-terminal sheet, where binding is seen in the other mentioned DOMON structures. This pocket in DBH is very leucine-rich. Several likely ligands could be ascorbate, fumarate, dopamine, or norepinephrine.

**Fig. 4:**
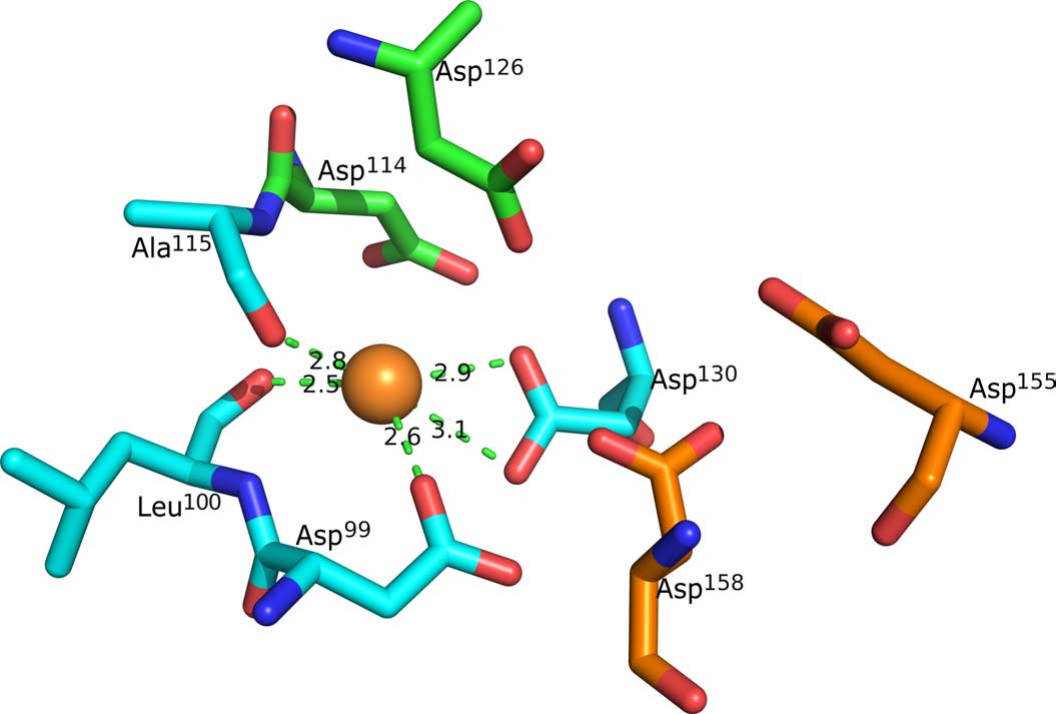
The putative metal ion–binding site in DBH DOMON. The prealigned coordinating residues (Asp^99^, Leu^100^, Ala^115^, and Asp^130^) are shown in blue. Other possible involved residues are shown in green and orange (Asp^114^, Asp^126^, Asp^155^, and Asp^158^). On the basis of the very oxygen-rich environment, it is likely to be either a group 1 or group 2 metal ion. The six mentioned aspartic acid residues are conserved among the DOMON domains in the copper-containing hydroxylases; see fig. S13.

Behind the possible ligand-binding pocket appears to be a metal ion–binding site coordinated by Asp^99^ OD1, Leu^100^ O, Ala^115^ O, and Asp^130^ OD1/OD2, with Asp^114^ and Asp^126^ quite close (see Fig. 4). These four aspartic acid residues, as well as two (Asp^155^ and Asp^158^) in the vicinity, are conserved among DBH DOMON domains from different organisms (see fig. S13). In the structure, we have placed water molecule 5 in chain A; however, in chain B, the electron density did not support modeling of an extra water molecule. On the basis of the very oxygen-rich ligand environment, it is likely to be either an alkali metal ion or an alkaline earth metal ion (group 1 or group 2 metal ion). However, a search using the CheckMyMetal server did not reveal the possible identity of the metal, most likely because not all the ligands are prealigned for metal binding.

The DBH DOMON domain is linked to the C-terminal part of the protein via a disulfide bridge between C^154^ and C^596^. It also contains two glycan sites at Asn^64^ and Asn^184^. Both of them could be built in chain A, whereas in chain B, the electron density only allowed for building of the glycan at Asn^64^.

### The catalytic core

The catalytic core consists of two domains: an N-terminal domain where Cu_H_ binds and a C-terminal domain where Cu_M_ binds. Both domains consist primarily of β sheets and have the approximate dimensions of 37 × 45 × 33 Å and 44 × 45 × 33 Å for chains A and B, respectively. Both domains have the same topology as described for PHM (*11*). The Cu_H_ domain folds into a β-sandwich formed of two antiparallel β sheets with four and five β-strands in each (see Fig. 1C). Strands β4 and β6 are held closely together with disulfide bridge C^269^-C^295^, and strands β2 and β5 are held together by disulfide bridge C^232^-C^283^. Furthermore, glycosylation is observed in the B chain at Asn^344^. The Cu_M_ domain folds into a β-sandwich containing a four-stranded antiparallel sheet and a five-stranded mixed sheet. The two sheets are held together by a very hydrophobic interior and two disulfide bridges connecting strands β8 and β9 (C^466^-C^488^) and strand β2 with β10 (C^390^-C^503^), respectively. The C-terminal sheet is actually a six-stranded sheet by the addition of a β-strand made of residues from the C terminus of the protein (residues 561 to 566) following the dimerization domain, as described above. The domain is further stabilized by a disulfide bridge to the additional strand C^394^-C^565^ (see Fig. 1, C and D). Finally, glycosylation is seen at Asn^366^ in both chains.

A comparison of the A and B chains shows that the Cu_H_ domain is positioned significantly different in the two chains, as shown in Fig. 2. As evident from Figs. 1 and 2, the Cu_H_ domain in chain A is moved away from the DOMON domain and closer to the Cu_M_ domain. This is also reflected in the domain-domain interactions listed in table S2.

Three of the four copper sites are not occupied, but the Cu_M_ site in chain A is weakly occupied, and a copper has been modeled. The weak occupancy of type 2 copper sites is an often encountered problem also seen in other copper enzymes like ceruloplasmin and laccase (*20*, *27*, *28*). Furthermore, the pH in the crystallization condition is approximately 4.2, well below the p*K*_a_ of the coordinating histidine residues, which therefore will be protonated and not able to coordinate copper. However, the approximate positions of the coppers are easily modeled from the positions of the conserved copper ligands His^262^, His^263^, and His^333^ for the Cu_H_ site and His^412^, His^414^, and Met^487^ for the Cu_M_ site. The copper ligands appear to be prealigned for Cu binding, except His^263^ in chain B, which is seen in a double conformation.

The copper-binding sites are located at the interface between the Cu_H_ and the Cu_M_ domains. It is seen that the interatomic distance between the copper ions is different in the two chains. The conformation in chain B is similar to what was observed in the structure of PHM (*11*), with a Cu-Cu distance of approximately 14 Å. However, as seen in Fig. 2C, in chain A the two copper ions are close together—approximately 4 to 5 Å in what appears to be a closed active site with a coupled binuclear copper site, as elaborated on below.

### The dimerization domain

**Fig. 5:**
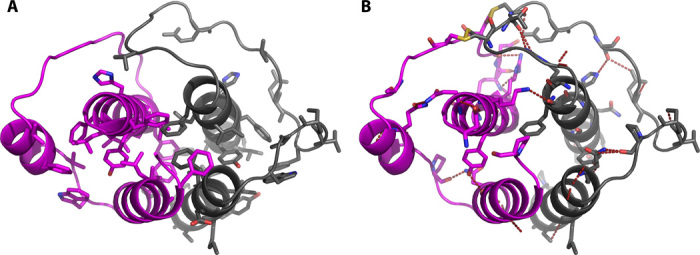
Domain interactions in the dimerization domain. Chain A is shown in magenta and chain B in gray. (**A**) Residues involved in hydrophobic interactions are shown as sticks. (**B**) Residues involved in hydrophilic interactions are shown as sticks, and the disulfide bridges are shown in yellow. Electron density maps for the disulfide bridges are provided in fig. S14.

The dimerization domain consists of two antiparallel α helices from each chain with quite long loop regions. It has been proposed on the basis of peptide mapping of bovine DBH that the domain should be held together by C^528^-C^528^ and C^530^-C^530^ disulfide bridges (*17*). However, we observe that the helices are definitely linked by C^528^A-C^530^B and C^530^A-C^528^B disulfide bonds. An electron density map is provided in fig. S14. The four-helix bundle is furthermore stabilized by both hydrophobic and electrostatic interactions, as shown in Fig. 5. The asymmetry of the overall structure is reflected in the dimerization domain, and creating an artificial dimer consisting of two open structures (a B-B dimer) shows that this is not possible because the chains would clash in the dimerization domain around residue 531. Surprisingly, the dimerization domain sequence (residues 508 to 560) has no sequence resemblance to any known domains (*16*), and a Dali server search did not reveal any closely related three-dimensional structures. However, the sequence is highly conserved among DBH from different organisms (see fig. S3).

The extra helix (residues 535 to 542) in the dimerization domain of molecule A makes hydrophobic interactions with residues 376 to 379 and 478 in the Cu_M_ domain. These are in close proximity to residues 481 to 483 stacking with the Cu_H_ domain in molecule A. However, the data do not allow for a detailed analysis of the hydrogen bond interactions.

The dimerization domain is followed by a long C-terminal extension with two β-strands. The first β-strand is part of the Cu_M_ domain, and the second β-strand is part of the DOMON domain (see Fig. 1 and fig. S9).

### Structural basis of DBH-related disorders

The presented crystal structure of DBH provides a structural framework for a better understanding of diseases involving DBH, as well as disease-causing mutations in DBH. DBH is implicated in many disorders, such as hypertension, congestive heart failure, depression, anxiety, Parkinson’s disease, Tourette syndrome, schizophrenia, and ADHD (*29*–*34*).

DBH is found in vesicles of central adrenergic and noradrenergic neurons, as well as peripheral noradrenergic neurons, and is released to the blood in response to stimulation [(*35*) and references therein]. The DBH activity level is stable within individuals but varies among individuals. In general, an association between lower plasma DBH activity and vulnerability to psychotic symptoms is observed (*9*). In patients suffering from norepinephrine deficiency, four potentially pathogenic mutations in the DBH gene have been identified (*29*). Norepinephrine deficiency is a congenital disorder in which the patients suffer profound autonomic failure. The following nonsynonymous coding region single-nucleotide polymorphisms (cSNPs) were identified in DBH: V101M, D114E, and D345N. The first two are positioned in the DOMON domain at (or next to) the suggested metal-binding site and at the bottom of the proposed ligand-binding pocket. These variants are therefore likely to influence both the metal-binding site and the ligand-binding site. D345N is a mutation in a long loop region between strand β9 and strand β10 in the Cu_H_ domain. However, the function of this residue is not obvious. Currently, 149 nonsynonymous cSNPs of human DBH are known [National Center for Biotechnology Information dbSNP (Single Nucleotide Polymorphism Database) Build 142], and they are distributed over the entire sequence; however, none of the copper ligands or the glycosylation sites are affected.

Inhibitors of DBH (nepicastat and etamicastat) are currently in clinical development for treatment of cocaine dependence (*8*). Posttraumatic stress disorder, hypertension, and heart failure (*36*), and also the structure of DBH described here, will facilitate further developments, because the inhibitor binding site and mode of action can be elucidated in detail.

## DISCUSSION

Here, we provide the first structure of DBH. It is tempting to speculate that the success in obtaining diffraction quality crystals is due to the more uniform glycosylation obtained in the HEK293S cells (*37*). We observe that human DBH heterologously expressed in HEK293S cells exists as a dimer and a tetramer, which do not interconvert. Likewise, no conversion is seen between the dimer and the tetramer of isolated native forms of human DBH (*38*) and human DBH expressed in *Drosophila* *melanogaster* cells (*39*), but in contrast, bovine DBH (*40*) interconverts between dimer and tetramer. We speculate that one reason for this difference could lie in the disulfide bridges in the dimerization domain. We observe linkages between C^528^A-C^530^B and C^528^B-C^530^A that are in contrast to what has been observed in the bovine enzyme, which is linked via C^528^-C^528^ and C^530^-C^530^. If the dimer-to-tetramer conversion requires a rearrangement of these disulfide bridges, it may explain why we do not observe interconversion. However, the dimer and tetramer interconversion could also be regulated by the still elusive possible ligand and metal ion that the structure suggests bind in the DOMON domain.

A comparison of the overall structure of the DBH monomers with the model structure published by Kapoor *et al.* (*16*) shows that it has, to a large extent, been possible to model the secondary structure elements of the DOMON domain and partially of the dimerization domain. From the illustrations, it seems that the overall model structure of the catalytic core corresponds to the open form (the B chain) that we have found. This is not surprising because the model proposed by Kapoor *et al.* is based on the PHM structures. It also appears that the overall topology (domain-domain orientation) of the monomer is different from our findings. The crystal structure also shows a much more integrated structure.

**Fig. 6:**
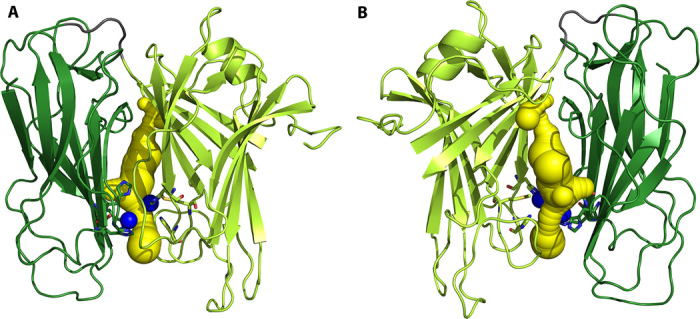
Binding pockets and channels in the vicinity of the closed active site seen in chain A. CAVER identified binding pocket and channel (yellow) in the closed catalytic core (chain A). The modeled Cu_M_ in the structure and the manually inserted copper ions are in blue. Two different orientations are shown. (**A**) Same orientation as in Fig. 2C, with the Cu_H_ domain to the left. (**B**) Viewed from the back, with the Cu_H_ domain to the right. Same color coding as in Fig. 1. The pocket is of sufficient size to hold the substrate (dopamine).

The two different conformations of the DBH catalytic core (shown in Fig. 2) offer different options for possible catalytic mechanisms, one interpretation being that the closed conformation seen in chain A is an artifact (stemming from, for example, the heterologous expression in HEK293 cells) and inactive, and that the conformation of chain B, which resembles the known structures of the PHM catalytic core, is the active form of the enzyme, with coppers 11 to 14 Å apart. In this case, the catalytic mechanism is, as previously described, for this enzyme family (*25*), which is supported by a number of structures of the PHM catalytic core (*11*) as well as spectroscopic and kinetic data [for reviews, see the studies by Osborne and Klinman (*25*) and Solomon *et al*. (*26*)]. In brief, the fully oxidized resting state is reduced by two molecules of ascorbic acid. Dioxygen then binds to Cu(I)_M_ that, together with substrate binding, gives a ternary complex from which dioxygen activation and substrate hydroxylation occur, involving an intramolecular electron transfer from Cu_H_ to Cu_M_. Finally, ascorbic acid binds to the substrate intermediate, triggering the release of the product. However, although a recent study on TBH has demonstrated a proton-coupled long-range electron transfer mechanism (*41*), one of the more difficult things to appreciate is how the necessary electron transfer proceeds over a distance of more than 10 Å with the lack of domain movement (*25*, *26*). The DBH structure indicates that maybe domain movement places the two copper sites close together during the electron transfer step. Yet another possible interpretation is that the closed site, seen in chain A, could in fact be the active site. With the short distance (4 to 5 Å) between the copper sites, the closed site resembles a coupled binuclear active site, which offers an appealing similarity to a number of other copper proteins that also process coupled binuclear copper sites, such as tyrosinase (EC 1.14.8.1) [for a recent review, see the study by Ramsden and Riley (*42*)], catechol oxidase (EC 1.10.3.1) (*43*), and the oxygen transport protein hemocyanin (*44*). In these proteins, the copper-copper distances vary from 2.2 to 4.9 Å (*26*). In the observed closed conformation in chain A, the possible active site is almost enclosed in the structure and shielded from its surroundings. Nevertheless, a CAVER search shows that there is room for the binding of a substrate in the closed conformation, as shown in Fig. 6. Currently, there is no other experimental evidence for a coupled binuclear site in DBH. A coupled binuclear copper site would, in most cases, be electron paramagnetic resonance–silent (*26*), but the anticipated active site intermediates during catalysis should have distinct spectroscopic signatures in electronic spectroscopy, x-ray absorption spectroscopy, and resonance Raman spectroscopy (*26*). More studies are required to illuminate this issue further.

**Fig. 7:**
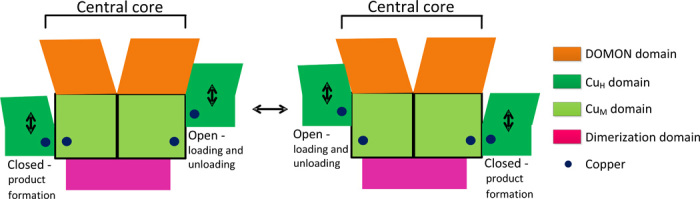
Proposed mode of action of DBH. The closed conformation with the coupled binuclear copper site is the catalytically active site. The open conformation serves as a way for loading of new substrate, release of product, and change in copper redox state. It is envisioned that the two sites alternate between the closed catalytically active form and the open form, known as a flip-flop mechanism (*45*, *46*).

On the basis of the presented structure of human DBH, a possible mode of action is that the closed form represents the catalytically active form, and that the open form is catalytically inactive but allows for loading of substrate, release of product, and recycling of the copper redox states. The two subunits then alternate between an open form and a closed catalytically active form (see Fig. 7). Other enzymes are known that have a similar mode of action, with changes between two different conformations, known as a flip-flop mechanism (*45*, *46*). One could further speculate that either the DOMON domain or the dimerization domain is controlling the conformational transition. However, PHM does not contain any of these domains, which indicates that conformational transition is an intrinsic property of this class of enzymes. The function of the DOMON domain could then be the allosteric regulation of the enzyme activity. However, additional studies are needed to further illuminate any of these issues.

In conclusion, we present the first structure of DBH, the enzyme converting the neurotransmitter dopamine to norepinephrine. The structure of the DOMON domain, a domain found in more than 1600 other proteins, reveals a possible metal-binding site and a possible ligand-binding pocket. The catalytic core structure shows both an open active site, similar to the structures of other enzymes in this family, and a closed active site, in which the two copper sites are only 4 to 5 Å apart, in what is best described as a coupled binuclear copper site. The dimerization domain adopts a conformation that bears no resemblance to any other known protein structure. Finally, the structure of DBH provides new insights into numerous devastating disorders associated with the dopamine system.

## MATERIALS AND METHODS

### Cloning and expression

The DNA coding sequence for soluble human DBH (GenBank accession no. P09172), residues 40 to 617 (for example, the first 39 residues being a signal anchor for type II membrane proteins were left out to obtain soluble DBH), was optimized for expression in HEK293 cells and synthesized by GenScript Corporation. For cloning into the pHLsec expression vector (*47*), the flanking sequences were modified by polymerase chain reaction (PCR). The forward primer introduces an Age I restriction enzyme site, whereas the reverse primer introduces a FLAG tag, two stop codons, and an Xho I site. The obtained PCR-modified gene was then cloned into the pHLsec vector by T4 ligase and transformed into *Escherichia* *coli* DH5α. Plasmid DNA for transfection was purified using GenElute HP Endotoxin-Free Plasmid Megaprep Kit from Sigma-Aldrich. Human DBH was then expressed by automated large-scale transient protein expression (*47*, *48*) in HEK293S GnTi^−^ cells (*37*).

### Purification

Cells were removed by centrifugation at 5000*g* and 4°C for 15 min, and the supernatant was filtered through a 0.22-μm filter. Protein from 1.5 liters of culture was purified at 4°C on a 5-ml anti-FLAG M2 affinity gel column equilibrated in 10 mM Hepes and 150 mM NaCl (pH 7.5). DBH was eluted with 5 mg of FLAG tag peptide per milliter in 10 mM Hepes and 150 mM NaCl (pH 7.5) and concentrated to 2 ml by ultrafiltration. DBH was present both as a tetramer and a dimer, which were separated on a Superdex 200 HR 16/60 in 10 mM Hepes and 150 mM NaCl (pH 7.5) at 1 ml/min. Fractions containing the dimeric DBH were collected and pooled; the final concentration that was also used for crystallization experiments was 6 mg/ml.

### SeMet protein preparation

SeMet labeled protein was expressed and purified as described above with the exception that the medium was exchanged every day and that the cells were harvested after 3 days. The yield was about 10% of that in normal medium.

### Crystallization

Glycosylated human DBH dimer FLAG-tagged and expressed in HEK293S GnTi^−^ cells was crystallized using the high-throughput facilities at Oxford Protein Production Facility (*49*). The condition was 15.2% polyethylene glycol 3350 (PEG3350) with 0.2 M potassium nitrate (pH 4.2). Crystals appeared after 1 day. The SeMet protein crystallized under the same conditions, except that 18.5% PEG3350 was used and the pH of the potassium nitrate was 4.6. SeMet crystals were stabilized by increasing the PEG concentration to 25%. The K_2_PtCl_4_ derivative was prepared by increasing the pH to 6.5 and soaking crystals for 3.5 hours at concentrations >20 mM. All crystals were transferred to a 25% (v/v) ethylene glycol/reservoir solution before flash-freezing them in liquid nitrogen.

### Data collection and structure determination

Diffraction data were collected at 100 K at the Diamond Light Source, beamlines I02 and I24. The following data sets were used in the structure determination: native 1, collected at I02 (λ = 1.0073 Å); native 2, collected at I24 (λ = 1.0071 Å); SeMet, collected at I24 (λ = 0.9789 Å); and K_2_PtCl_4_ (λ = 1.0714 Å), also collected at I24. All data were processed with xia2 (*50*)/XDS (*51*). The structure was determined by a combination of multiple isomorphic replacement/multiwavelength anomalous dispersion and molecular replacement: at first, 15 selenium positions were determined with hkl2map (*52*), and then, electron density maps were calculated with autosharp (*53*) using native 2, SeMet, and K_2_PtCl_4_ data sets. This map was of sufficient quality to place two molecules of 1OPM (*54*) into the density with molrep (*55*)/CCP4i (*56*). Subsequently, several cycles of phasing and model building with phenix.autosol (*57*) were performed using native 1 and SeMet data. This revealed a domain movement in one of the two molecules (molecule A), and the resulting maps allowed a detailed model to be built.

The DOMON and the C-terminal domains were built manually in Coot because all attempts to perform automatic building failed. Met^89^ was used as a marker for the sequence together with the glycosylated Asn^64^ with the neighboring Trp^63^. In the catalytic domains, the amino acid sequence was changed according to a structural alignment. The initial refinement was performed in refmac5 (*58*); later, phenix.refine (*57*) was used. Noncrystallographic symmetry between the individual domains and torsion/libration/screw (TLS) motion were applied. The TLS domains in the two chains were defined differently using the TLS server (*59*). Chain A was divided as follows: 46 to 78, 79 to 187, 188 to 236, 237 to 362, 363 to 523, 524 to 576, and 577 to 611, and molecule B was divided as follows: 47 to 187, 188 to 523, and 524 to 612. Glycosylation sites were filled in as they appeared in the difference density. In subunit A, the Cu_M_ atom was inserted—it is weakly occupied, though, and could just as well be modeled with a water molecule. All together, only 13 water molecules were inserted on the basis of the difference density. Some parts of the structure could not be modeled because of missing or inadequate electron density. The following residues were missing: A^40^ to A^45^, A^109^, A^593^ to A^607^, and A^612^ to A^617^; and B^40^ to B^46^, B^104^ to B^109^, B^273^ to B^275^, B^288^ to B^291^, B^597^ to B^608^, and B^615^ to B^617^. The quality of the final structure was evaluated with MolProbity (*60*). A total of 99.5% of the residues were in Ramachandran favored or allowed regions. The statistics of the data collection and refinement are summarized in table S3.

### Structural analysis

All molecular graphics were prepared using PyMOL (*61*). Protein alignments were done using the Clustal Omega (*62*) and the CLC Main Workbench 7.5. For the analysis, the following servers and plug-ins were used: Dali (*63*), CASTp (*64*), CheckMyMetal (*65*), and CAVER (*66*).

## Supplementary Material

http://advances.sciencemag.org/cgi/content/full/2/4/e1500980/DC1

## SUPPLEMENTARY MATERIALS

Supplementary material for this article is available at http://advances.sciencemag.org/cgi/content/full/2/4/e1500980/DC1 Supplementary Materials and Methods Fig. S1. Overall domain alignment of copper-containing hydroxylases. Fig. S2. Sequence alignment of copper-containing hydroxylases. Fig. S3. Sequence alignment of DBH from different organisms. Fig. S4. Size exclusion analysis of purified DBH tetramer and dimer. Fig. S5. Analysis of DBH tetramer conversion as a function of pH. Fig. S6. Analysis of DBH tetramer conversion as a function of ionic strength. Fig. S7. Mass spectrum of a nonseparated sample containing a mixture of dimeric and tetrameric DBH. Fig. S8. SDS–polyacrylamide gel electrophoresis analysis of dimeric and tetrameric DBH under nonreducing and reducing conditions. Fig. S9. Structure of the human DBH dimer emphasizing the integrated structure created by the C-terminal interaction with both the Cu_M_ domain and the DOMON domain. Fig. S10. Modeled glycosylation environments in chain A with 2*F*_obs_ − *F*_calc_ electron density maps contoured at σ of 1.0. Fig. S11. Modeled glycosylation environments in chain B with 2*F*_obs_ − *F*_calc_ electron density maps contoured at σ of 1.0. Fig. S12. Structure of the human DBH dimer with the disulfide bridges and the glycosylation sites highlighted. Fig. S13. Sequence alignment of DOMON domains. Fig. S14. The dimerization domain disulfide bridges environment with 2*F*_obs_ − *F*_calc_ electron density map contoured at σ of 1.0. Table S1. Secondary structure assignment in human DBH. Table S2. Domain-domain hydrogen bond contacts in chains A and B. Table S3. Data collection, phasing, and refinement statistics.
