# Supplementary material for: The crystal structure of human dopamine β-hydroxylase at 2.9 Å resolution
Source: Sci Adv. 2016 Apr 8;2(4):e1500980. doi: 10.1126/sciadv.1500980 (PMC4846438; doi:10.1126/sciadv.1500980)
Supplement: http://advances.sciencemag.org/cgi/content/full/2/4/e1500980/DC1 [file supp_2_4_e1500980__index.html]

Science Advances | Science Advances

## Supplementary Materials

**This PDF file includes:**

- Supplementary Materials and Methods
- Fig. S1. Overall domain alignment of copper-containing hydroxylases.
- Fig. S2. Sequence alignment of copper-containing hydroxylases.
- Fig. S3. Sequence alignment of DBH from different organisms.
- Fig. S4. Size exclusion analysis of purified DBH tetramer and dimer.
- Fig. S5. Analysis of DBH tetramer conversion as a function of pH.
- Fig. S6. Analysis of DBH tetramer conversion as a function of ionic strength.
- Fig. S7. Mass spectrum of a nonseparated sample containing a mixture of dimeric and tetrameric DBH.
- Fig. S8. SDS–polyacrylamide gel electrophoresis analysis of dimeric and tetrameric DBH under nonreducing and reducing conditions.
- Fig. S9. Structure of the human DBH dimer emphasizing the integrated structure created by the C-terminal interaction with both the CuM domain and the DOMON domain.
- Fig. S10. Modeled glycosylation environments in chain A with 2*F*obs − *F*calc electron density maps contoured at σ of 1.0.
- Fig. S11. Modeled glycosylation environments in chain B with 2*F*obs − *F*calc electron density maps contoured at σ of 1.0.
- Fig. S12. Structure of the human DBH dimer with the disulfide bridges and the glycosylation sites highlighted.
- Fig. S13. Sequence alignment of DOMON domains.
- Fig. S14. The dimerization domain disulfide bridges environment with 2*F*obs − *F*calc electron density map contoured at σ of 1.0.
- Table S1. Secondary structure assignment in human DBH.
- Table S2. Domain-domain hydrogen bond contacts in chains A and B.
- Table S3. Data collection, phasing, and refinement statistics.

Download PDF

**Files in this Data Supplement:**

- Adobe PDF - 1500980\_SM.pdf
