# Supplementary material for: The crystal structure of human dopamine β-hydroxylase at 2.9 Å resolution
Source: Sci Adv. 2016 Apr 8;2(4):e1500980. doi: 10.1126/sciadv.1500980 (PMC4846438; doi:10.1126/sciadv.1500980)
Supplement: http://advances.sciencemag.org/cgi/content/full/2/4/e1500980/DC1 [file 1500980_SM.pdf]

Science Advances

AAAS

advances.sciencemag.org/cgi/content/full/2/4/e1500980/DC1

Supplementary Materials for

The crystal structure of human dopamine β-hydroxylase at 2.9 Å resolution

Trine V. Vendelboe, Pernille Harris, Yuguang Zhao, Thomas S. Walter, Karl Harlos, Kamel El Omari, Hans E. M. Christensen

Published 8 April 2016, *Sci. Adv.* **2**, e1500980 (2016)
DOI: 10.1126/sciadv.1500980

The PDF file includes:

- Supplementary Materials and Methods
- Fig. S1. Overall domain alignment of copper-containing hydroxylases.
- Fig. S2. Sequence alignment of copper-containing hydroxylases.
- Fig. S3. Sequence alignment of DBH from different organisms.
- Fig. S4. Size exclusion analysis of purified DBH tetramer and dimer.
- Fig. S5. Analysis of DBH tetramer conversion as a function of pH.
- Fig. S6. Analysis of DBH tetramer conversion as a function of ionic strength.
- Fig. S7. Mass spectrum of a nonseparated sample containing a mixture of dimeric and tetrameric DBH.
- Fig. S8. SDS–polyacrylamide gel electrophoresis analysis of dimeric and tetrameric DBH under nonreducing and reducing conditions.
- Fig. S9. Structure of the human DBH dimer emphasizing the integrated structure created by the C-terminal interaction with both the Cu_M domain and the DOMON domain.
- Fig. S10. Modeled glycosylation environments in chain A with $2F_{obs} - F_{calc}$ electron density maps contoured at σ of 1.0.
- Fig. S11. Modeled glycosylation environments in chain B with $2F_{obs} - F_{calc}$ electron density maps contoured at σ of 1.0.
- Fig. S12. Structure of the human DBH dimer with the disulfide bridges and the glycosylation sites highlighted.
- Fig. S13. Sequence alignment of DOMON domains.
- Fig. S14. The dimerization domain disulfide bridges environment with $2F_{obs} - F_{calc}$ electron density map contoured at σ of 1.0.
- Table S1. Secondary structure assignment in human DBH.

- Table S2. Domain-domain hydrogen bond contacts in chains A and B.
- Table S3. Data collection, phasing, and refinement statistics.

SUPPLEMENTARY MATERIALS AND METHODS

Mass spectrometry analysis and sample preparation

Native protein mass spectrometry analysis was carried out on a LCT premier instrument with a nano-ESI source (Waters). The spectrometer was equilibrated with 100 mg/ml CsI in 50 % 2-propanol. The spectra were recorded in positive ion mode, with a cone voltage of 50-200 V, an ion guide I voltage of 175 V and a capillary voltage of 1.5 – 1.75 x 10^3 V.

DBH samples at different pH and ionic strength were prepared by buffer exchange using Micro Bio-Spin 6 Chromatography columns (Bio-Rad). The columns were prepared with ammonium acetate at various concentrations and pH values. The ammonium acetate solutions were prepared from a 7.5 M ammonium acetate stock solution in plastic bottle (from Sigma Aldrich). The pH adjustments were done with diluted ammonia or acetic acid in plastic containers in order to avoid sodium ion contamination from glassware. Samples were incubated at least 1 h at room temperature before analysis.

Size exclusion analysis

The analyses were performed on a Superdex 200 10/300 column in 10 mM hepes, 150 mM NaCl, pH 7.5 at a flow rate of 0.5 ml/min using an ÄKTA purifier (GE Healthcare, USA). The absorbance was measured at 280 nm.

SDS-Page analysis

Sodium dodecyl sulfate–polyacrylamide gel electrophoresis (SDS-Page) analysis was carried out according to manufacturer's recommendations, using a precast gel and markers from Bio-Rad. The gel is stained with Coomassie blue G-250.

FIGURES AND TABLES

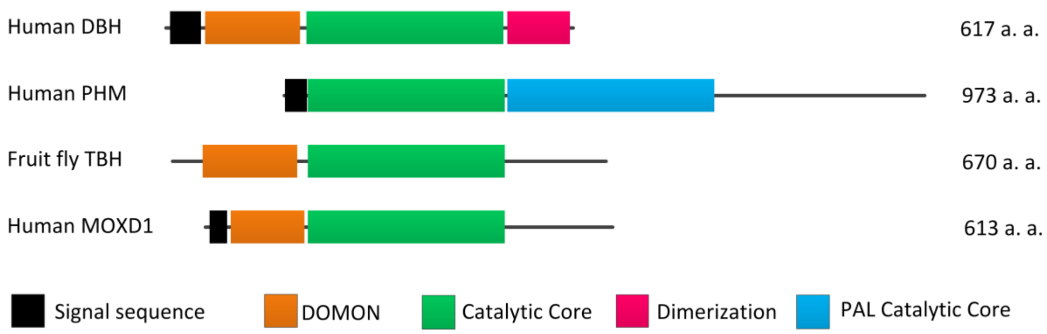

fig. S1. Overall domain alignment of copper-containing hydroxylases. DBH, dopamine β-hydroxylase; PHM, peptidylglycine α-hydroxylating monooxygenase; TBM, tyramine β-mono-oxygenase and MOXD1, monooxygenase X. DOMON, dopamine β-hydroxylase N-terminal domain; PAL, peptidyl-α-hydroxyglycine α-amidating lyase. Amino acid sequence alignments are provided in fig. S2.

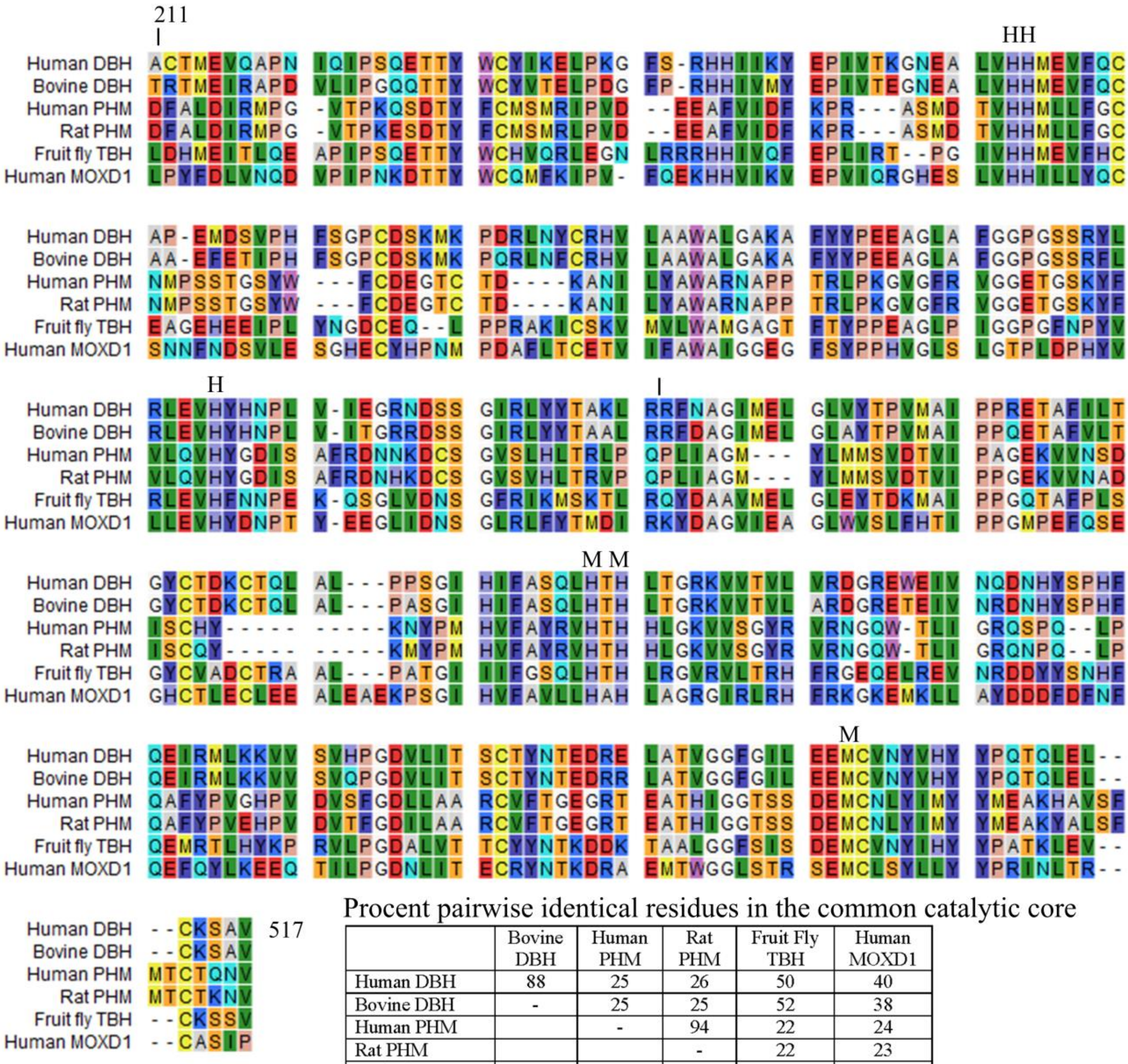

Procent pairwise identical residues in the common catalytic core

	Bovine DBH	Human PHM	Rat PHM	Fruit Fly TBH	Human MOXD1
Human DBH	88	25	26	50	40
Bovine DBH	-	25	25	52	38
Human PHM		-	94	22	24
Rat PHM			-	22	23
Fruit Fly TBH				-	37

fig. S2. Sequence alignment of copper-containing hydroxylases. Human DBH (UniProt id P09172, residue 211-507), bovine (*Bos taurus*) DBH (UniProt id P15101, residue 204-500), human PHM (UniProt id P19021, residue 56-333), rat (*Rattus norvegicus*) PHM (UniProt id P14925, residue 61-338), fruit fly (*Drosophila melanogaster*) TBM (UniProt id Q86B61, residue 262-555) and human MOXD1 (UniProt id Q6UVY6, residue 184-484). Cu_H and Cu_M ligands are labelled H and M, respectively. | indicates the boundary between the Cu_H domain and the Cu_M domain.

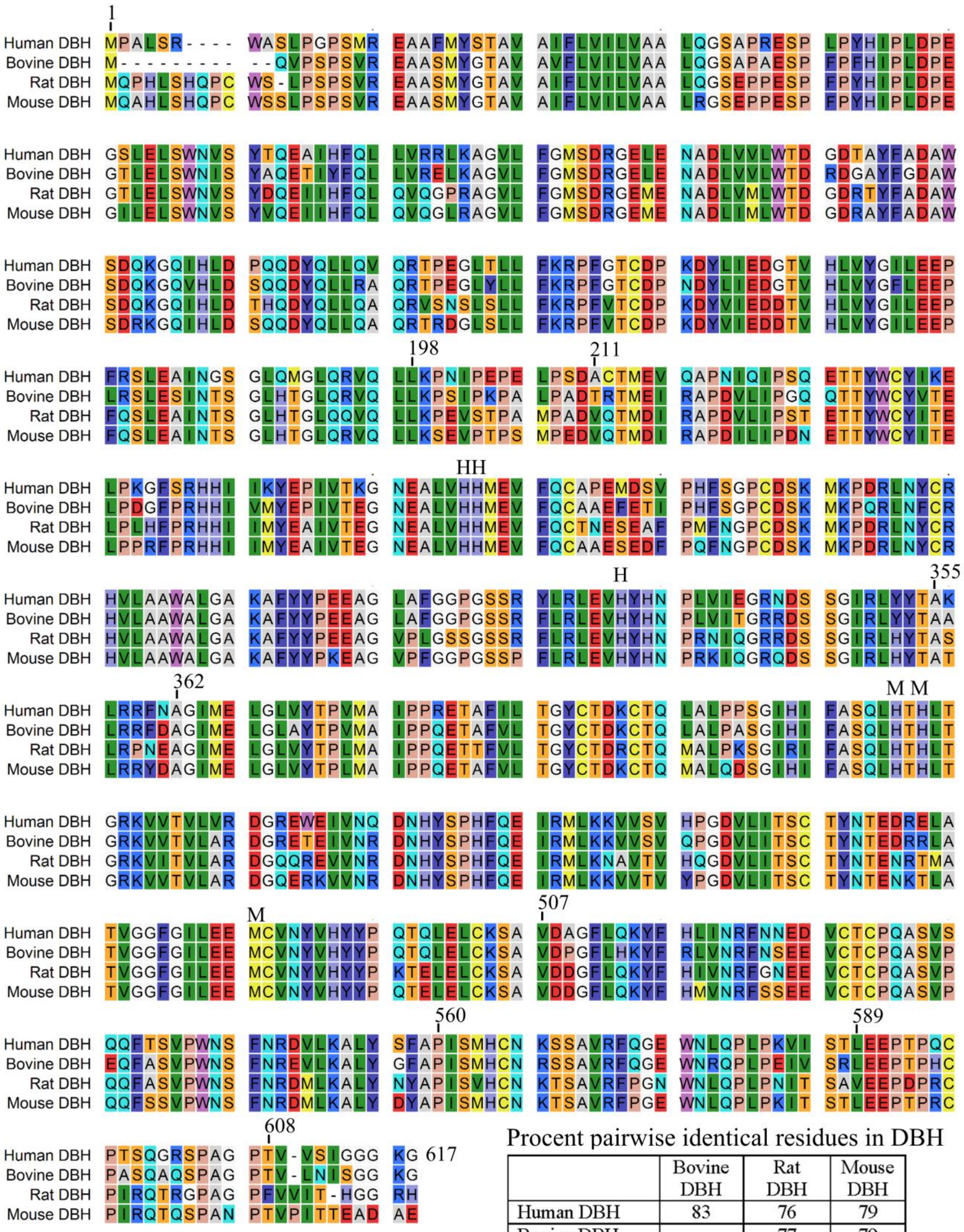

Procent pairwise identical residues in DBH

	Bovine DBH	Rat DBH	Mouse DBH
Human DBH	83	76	79
Bovine DBH	-	77	79
Rat DBH		-	88

fig. S3. Sequence alignment of DBH from different organisms. Human DBH (UniProt id P09172), bovine (*Bos taurus*) DBH (UniProt id P15101), rat (*Rattus norvegicus*) DBH (UniProt id Q05754) and mouse (*Mus musculus*) DBH (UniProt id Q64237). Cu_H and Cu_M ligands are labelled H and M, respectively. 14 of 15 cysteine residues (in yellow) are conserved among organisms (Cys^{212} is not conserved).

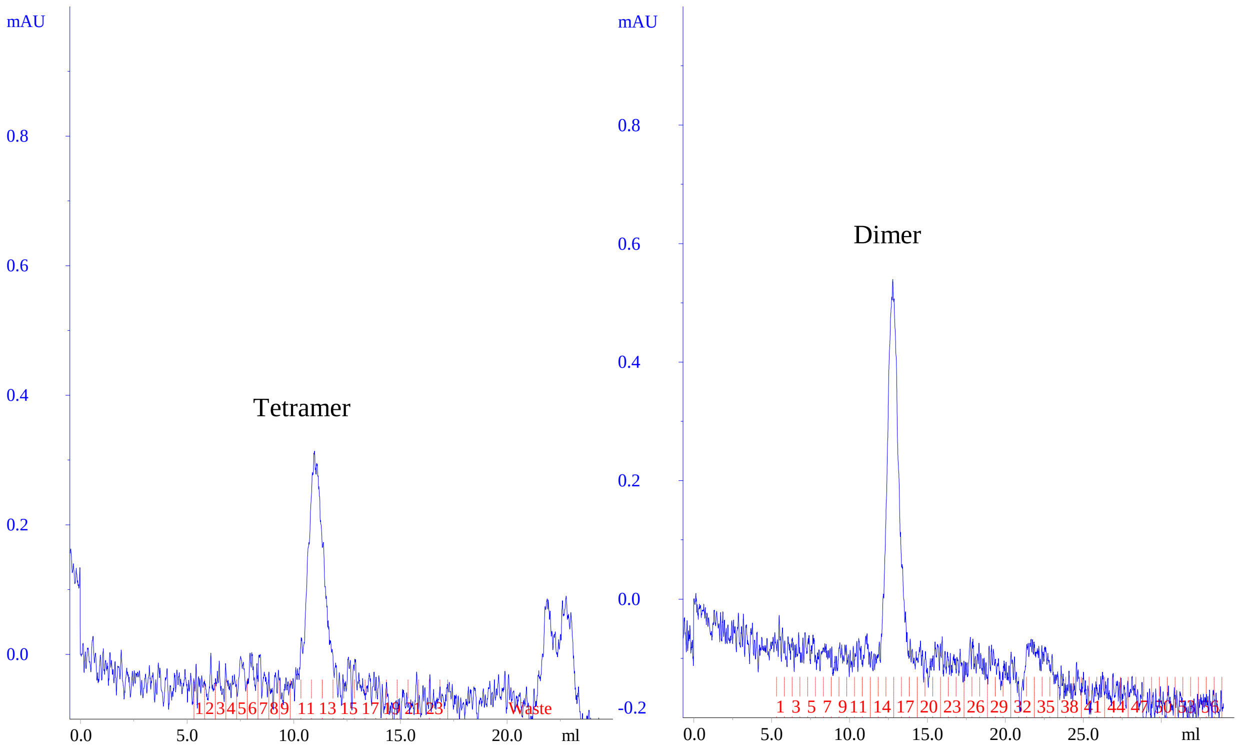

fig. S4. Size exclusion analysis of purified DBH tetramer and dimer. To the left analysis of purified DBH tetramer. To the right analysis of purified DBH dimer. The absorbance at 280 nm is shown in blue. In both cases no conversion to the other form is seen, showing that in the purification buffer (10 mM Hepes, 150 mM NaCl, pH 7.5) both the tetramer and the dimer are stable.

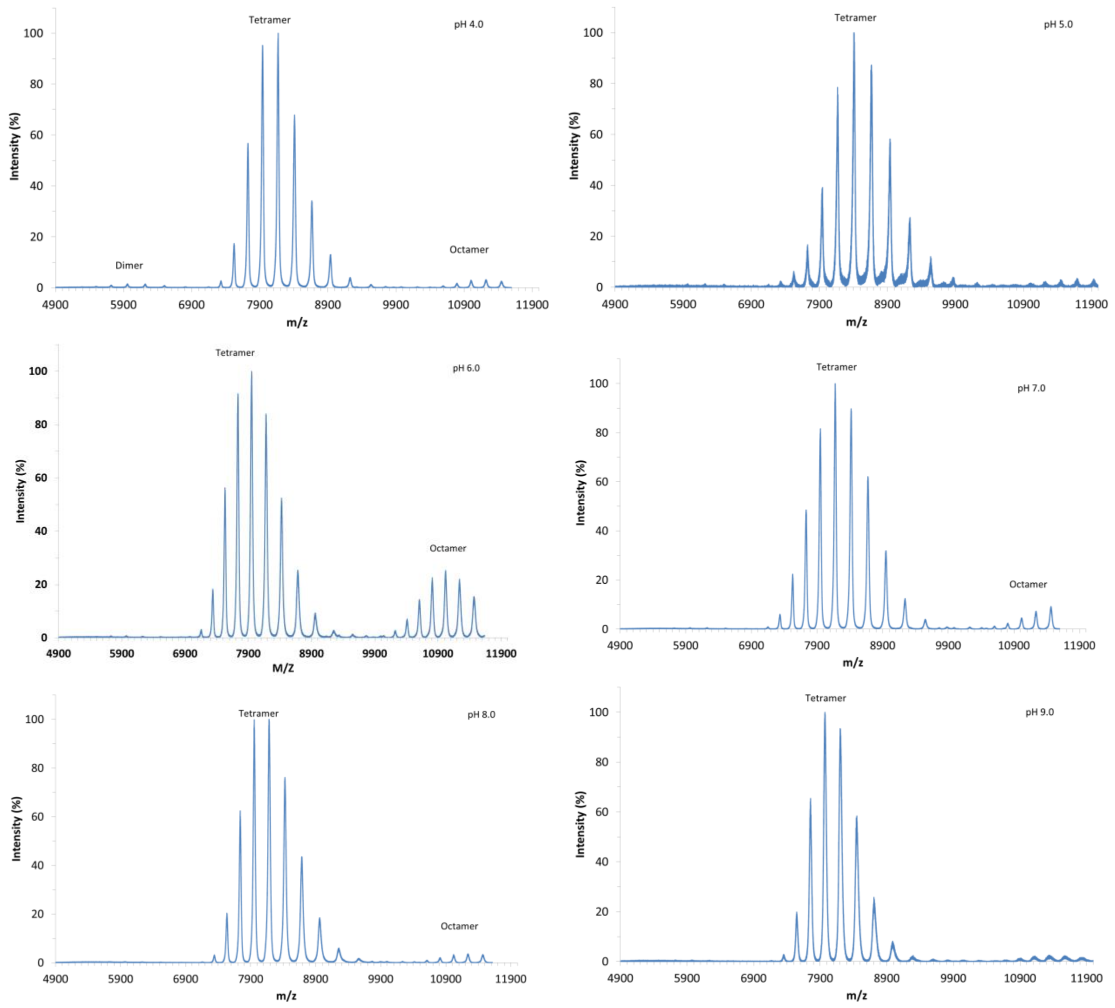

fig. S5. Analysis of DBH tetramer conversion as a function of pH. Samples of DBH tetramer were incubated in the pH interval 4 to 9 for 1 hour and analyzed by mass spectrometry. Only the envelop corresponding to the DBH tetramer is observed, no dimer is seen. In some samples small quantities of octamer DBH is observed, which is considered an artefact from the MS analysis.

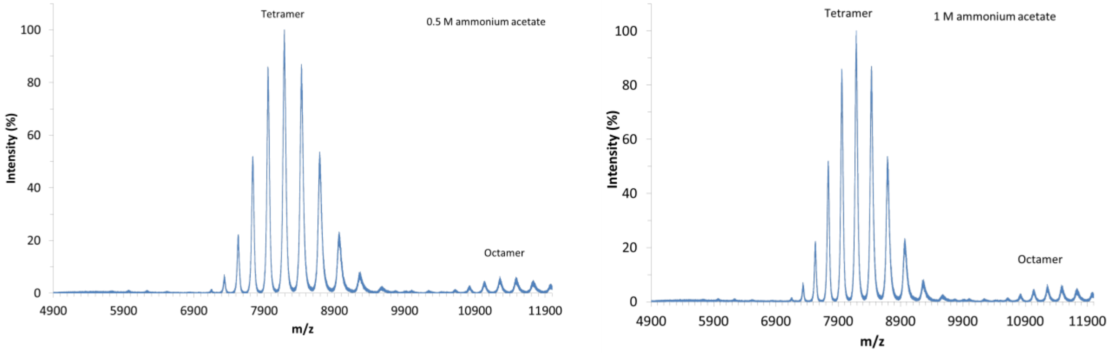

fig. S6. Analysis of DBH tetramer conversion as a function of ionic strength. Samples of DBH tetramer were incubated at ionic strength 0.5 M and 1M at pH 6 and analyzed by mass spectrometry. Only the envelop corresponding to the DBH tetramer is observed, no dimer is seen.

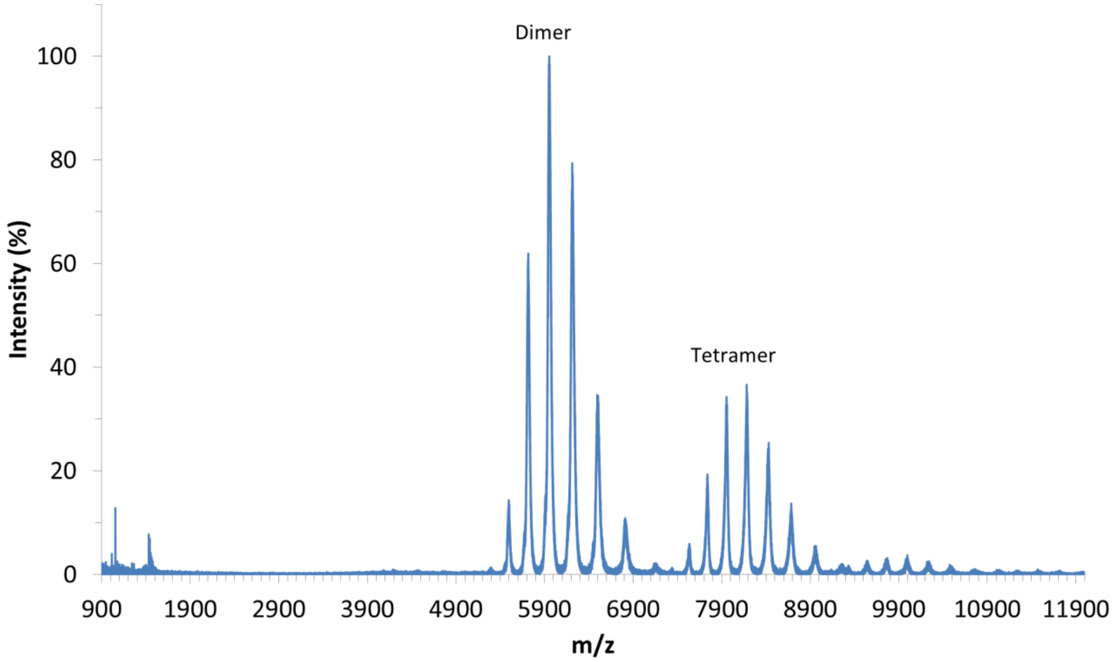

fig. S7. Mass spectrum of a nonseparated sample containing a mixture of dimeric and tetrameric DBH. The sample is 15 µM DBH in 0.5 M ammonium acetate. Both tetramer DBH and dimer DBH are observed, confirming that both dimeric and tetrameric DBH are easily observed if presence.

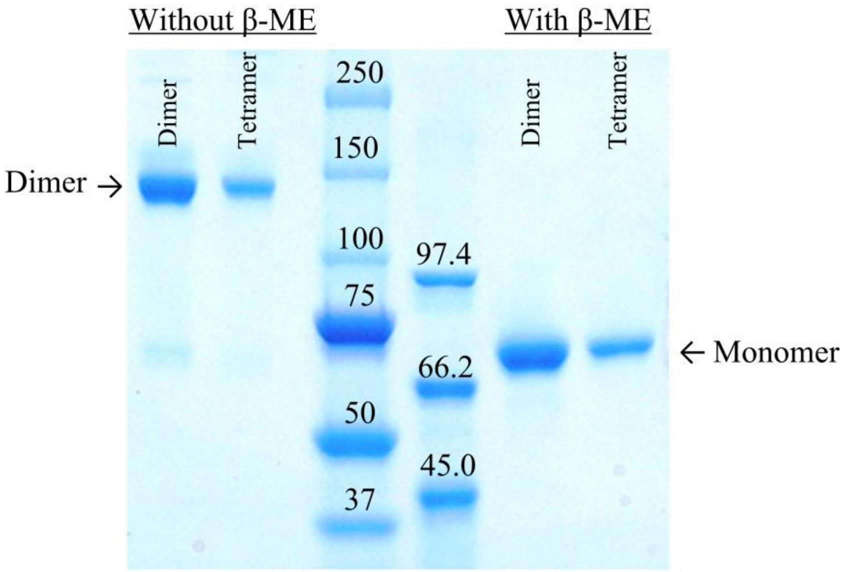

fig. S8. SDS–polyacrylamide gel electrophoresis analysis of dimeric and tetrameric DBH under nonreducing and reducing conditions. Under non-reducing conditions (to the left) dimeric and tetrameric DBH have the same mass corresponding to a dimer of ~150 kDa. To the right under reducing conditions (with β-mercaptoethanol) dimeric and tetrameric DBH both have the mass corresponding to a monomer of ~70 kDa. Markers (given in kDa) are Precision Plus Protein Standards and Low Range Standards from Bio-Rad. The SDS-Page analysis reveals that the dimer of dimers in the tetramer are not covalently bound, as no reducing agent is required to separate the tetramer into dimer under denaturation conditions.

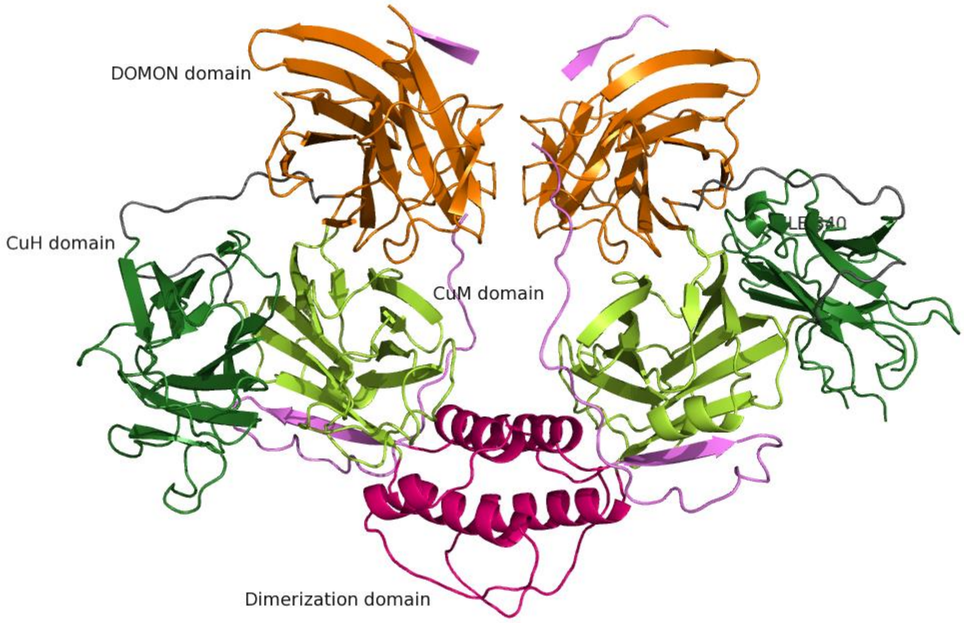

fig. S9. Structure of the human DBH dimer emphasizing the integrated structure created by the C-terminal interaction with both the Cu$_M$ domain and the DOMON domain. The C-terminus is in violet.

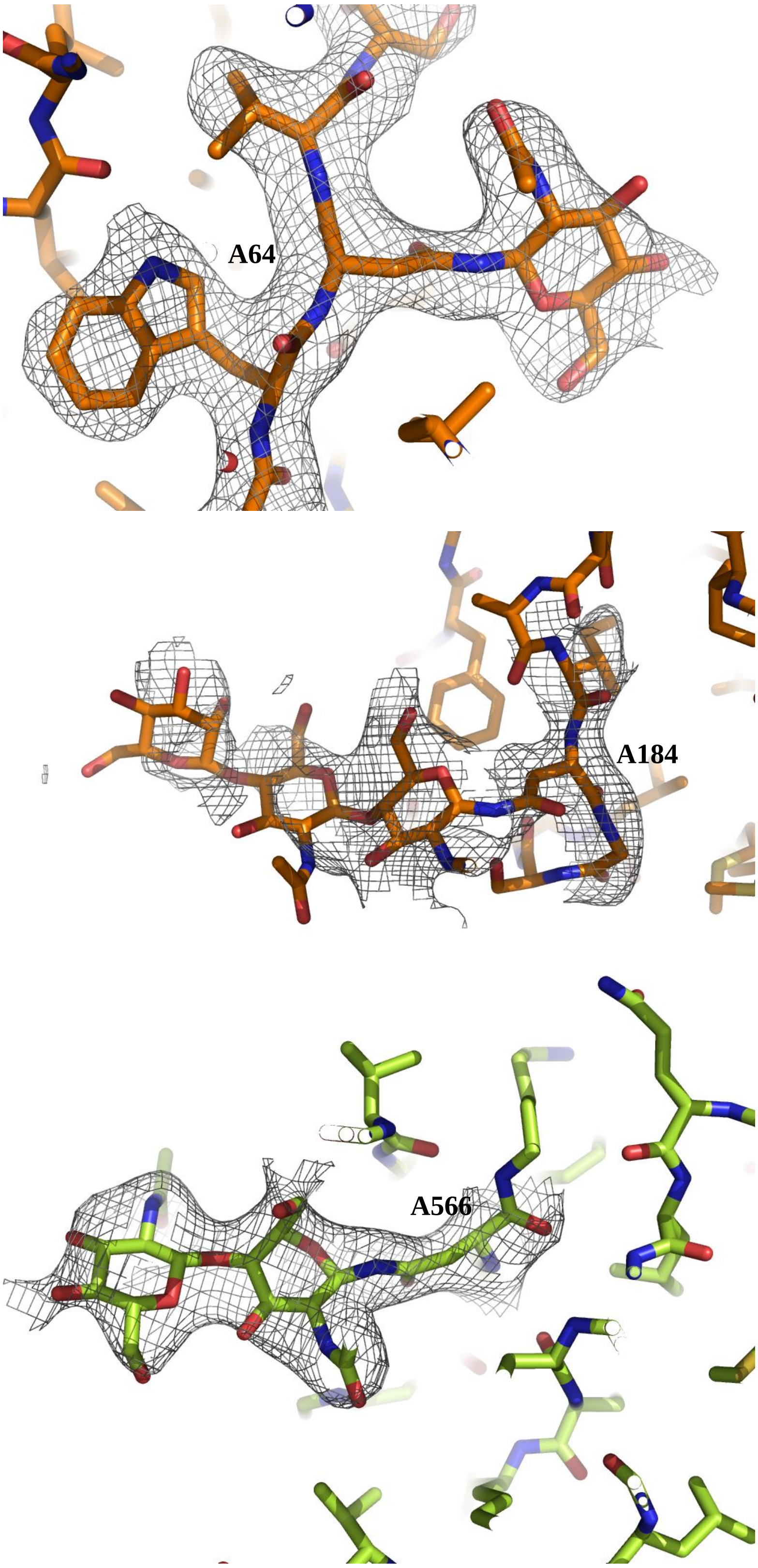

fig. S10. Modeled glycosylation environments in chain A with $2F_{obs} - F_{calc}$ electron density maps contoured at σ of 1.0. Colored in domain colors (same color scheme as in Fig. 1 and Fig. 2) and with oxygen in red and nitrogen in blue.

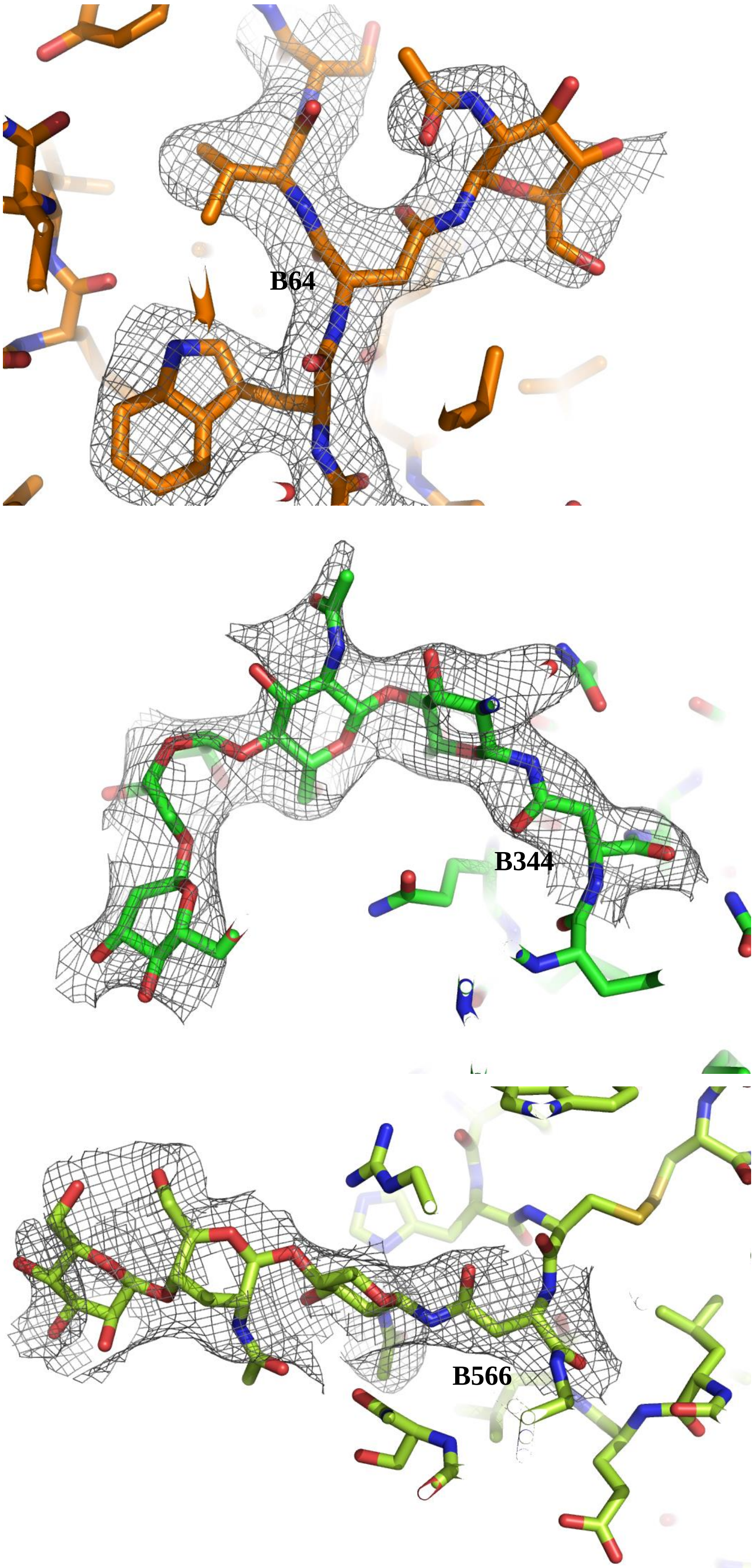

fig. S11. Modeled glycosylation environments in chain B with $2F_{obs} - F_{calc}$ electron density maps contoured at σ of 1.0. Colored in domain colors (same color scheme as in Fig. 1 and Fig. 2) and with oxygen in red and nitrogen in blue.

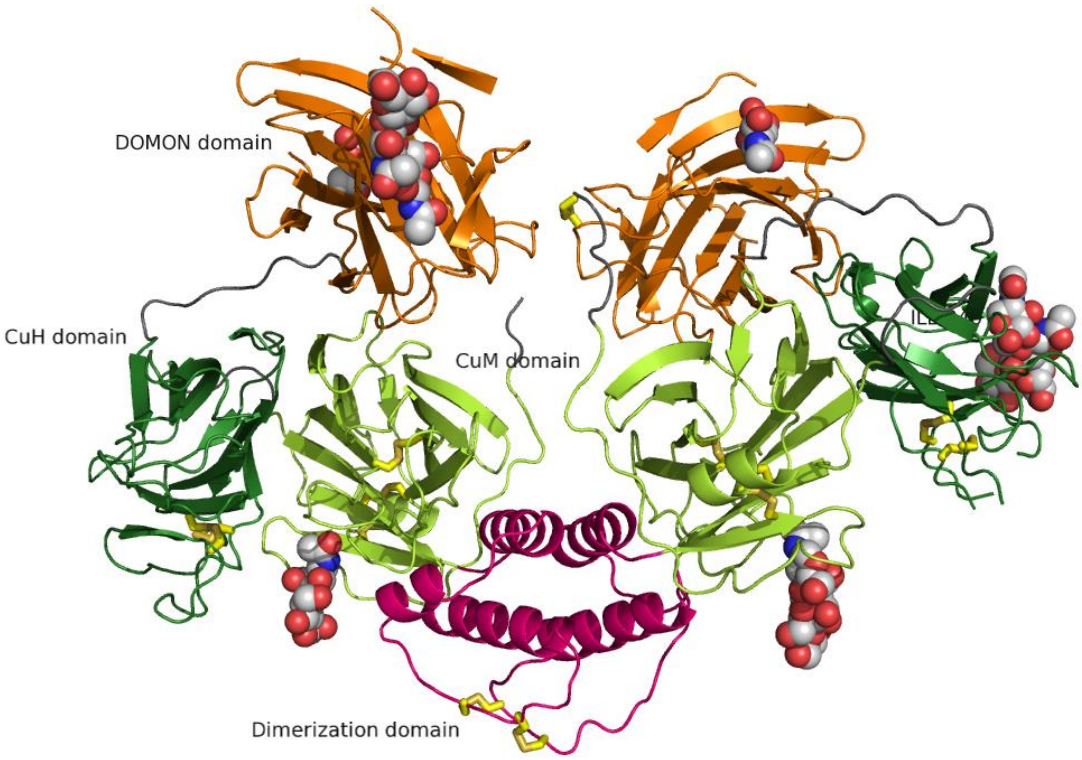

fig. S12. Structure of the human DBH dimer with the disulfide bridges and the glycosylation sites highlighted. The disulfide bridges are in yellow and the modeled glycosylation is shown as CPK's.

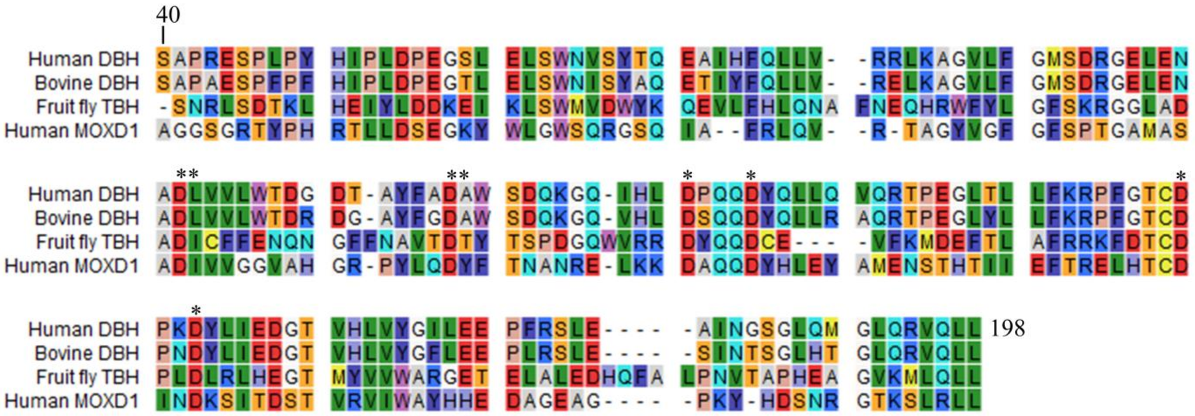

Procent pairwise identical residues in the DOMON domain

	Bovine DBH	Fruit Fly TBH	Human MOXD1
Human DBH	85	24	28
Bovine DBH	-	25	28
Fruit Fly TBH		-	21

fig. S13. Sequence alignment of DOMON domains. Human DBH (UniProt id P09172, residue 40-198), bovine (*Bos taurus*) DBH (UniProt id P15101, residue 33-191), fruit fly (*Drosophila melanogaster*) TBM (UniProt id Q86B61 residue 88-251) and human MOXD1 (UniProt id Q6UVY6 residue 18-173). Residues involved in the putative metal binding site are labeled *, see main text and Fig. 4.

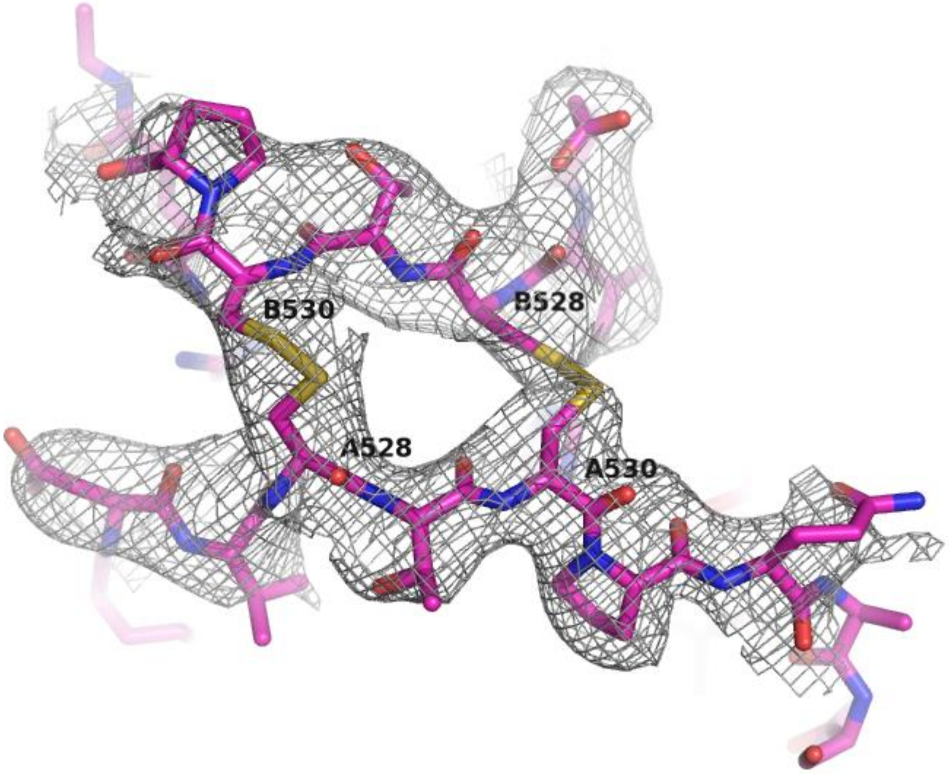

fig. S14. The dimerization domain disulfide bridges environment with 2F_{obs} – F_{calc} electron density map contoured at σ of 1.0. The disulfide bridges are colored yellow and with oxygen in red and nitrogen in blue.

table S1. Secondary structure assignment in human DBH.

**DOMON domain**	A-chain	B-chain
β1	49-52	49-52
β2	59-66	59-66
β3	71-78	71-78
β4	84-90	84-90
β5	98-104	95-103
β6	111-117	113-117
β7	123-125	123-125
β8	132-139	131-138
β9	143-150	143-150
β10	166-173	165-173
β11	191-195	188-195
C-term β2	608-611	609-611
**Cu_H domain**		
β1	212-217	212-217
β2	228-236	228-236
β3	244-252	244-252
β4	261-268	261-268
β5	279-282	280-282
β6	297-303	298-303
β7	308-310	308-310
β8	317-319	317-319
β9	327-335	327-335
β10	348-354	348-354
**Cu_M domain**		
β1	363-369	363-369
β2	384-390	384-390
α1	392-399	393-398
β3	404-412	404-413
β4	417-425	417-425
β5	432-438	432-437
β6	445-449	447-449
β7	454-456	454-456
β8	461-468	461-468
β9	489-495	488-495
β10	502-507	502-507
C-term β1	561-567	561-567
**Dimerization domain**		
α1	509-523	509-523
α*	535-539	
α2	546-558	546-558

table S2. Domain-domain hydrogen bond contacts in chains A and B. In the A-chain there are 10 hydrogen bonds between the Cu_H domain and the Cu_M domain. In the B-chain the contacts are reduced to only 4 hydrogen bonds. Between the DOMON domain and the Cu_H domain there are no contacts in the A-chain while in the B-chain some non-hydrogen bond interactions are present.

	Chain A	Å	Chain B	Å
Cu_H domain with Cu_M domain	G316 O – A362 N	2.9	Y310 N – M449 SD	3.5
	L304 O – H443 O	3.5	E314 OE1 – Y495 OH	3.2
	L304 O – Q445 N	2.7	E314 OE2 – Y495 OH	3.5
	L304 O – Q445 O	2.9	G316 O – A362 N	3.1
	A306 N – Q445 O	3.1		
	Y230 OH – F481 O	2.3		
	H297 NE2 – Q497 NE2	3.5		
	R296 O – E501 OE1	2.6		
	H297 N – E501 OE1	3.4		
	V298 N – E501 OE2	2.9		

table S3. Data collection, phasing, and refinement statistics*.

	Native-1	Native-2	SeMet	K_2PtCl_4
**Data collection**				
Space group	$C222_1$	$C222_1$	$C222_1$	$C222_1$
Cell dimensions				
a, b, c (Å)	102.8, 119.1, 224.8	102.4, 118.8, 225.2	102.5, 119.0, 225.5	103.0, 119.8, 225.4
α, β, γ (°)	90, 90, 90	90, 90, 90	90, 90, 90	90, 90, 90
			Peak	*Peak*
Wavelength	1.0073	1.0071	0.9789	1.0714
Resolution (Å)	63.98-2.90 (2.98-2.90)	53.43-2.67 (2.74-2.67)	56.37-3.08 (3.11-3.08)	57.88-3.83 (3.93-3.83)
R_{sym} or R_{merge}	0.147 (1.263)	0.300 (3.738)	0.253 (0.489)	0.405 (3.019)
I/σ_I	10.6 (1.6)	10.6 (1.1)	11.7 (1.7)	6.4 (0.9)
Completeness (%)	99.9 (99.8)	65.5 (19.4)	92.3 (46.7)	96.1 (54.1)
Redundancy	6.5 (6.9)	16.0 (9.5)	11.2 (1.7)	11.2 (8.7)
**Refinement**				
Resolution (Å)	63.9-2.90			
No. of reflections	30957 (223)			
R_{work}/ R_{free}	0.232/ 0.270			
No. atoms				
Protein	8945			
Ligand/ion	1			
Water	14			
B-factors				
Protein	75.1			
Cu-ion	162.1			
Water	42.4			
R.m.s deviations				
Bond lengths (Å)	0.007			
Bond angles (°)	1.50			

Native-1 and 2 were collected with a single crystal. For SeMet and K_2PtCl_4 three crystals were used.
*Highest resolution shell is shown in parenthesis.
